# The Implementation of Robotics in the Setting of Hiatal Hernia Repair: A Scoping Review on Current Evidence

**DOI:** 10.7759/cureus.99088

**Published:** 2025-12-13

**Authors:** Dimitrios G Armamentos, Dimitrios Xenofos, Nikoleta Sinou, Natalia Sinou, Dimitrios Filippou

**Affiliations:** 1 Anatomy, National and Kapodistrian University of Athens School of Medicine, Athens, GRC; 2 Medicine, Research and Education in Biomedical Sciences (REIBS), Piraeus, GRC

**Keywords:** clinical outcome, efficacy, hiatal hernia, laparoscopy, repair, robotic surgery

## Abstract

Background and objective:For many years, laparoscopy has been recognized as the standard approach for treating hiatal hernia (HH). Nevertheless, it continues to face significant recurrence rates and specific technical challenges. With the swift advancement of technology and the gradual adoption of robotic systems in clinical practice, robotic HH repair has generated interest due to its potential advantages, including three-dimensional visualization, improved dexterity, and better ergonomics. This scoping review seeks to assess the safety, feasibility, and effectiveness of robotic HH repair.

Material and methods: A thorough literature search was performed in the PubMed and Scopus databases between August 13 and October 1, 2025, adhering to the Preferred Reporting Items for Systematic reviews and Meta-Analyses extension for Scoping Reviews (PRISMA-ScR) criteria and utilizing the keywords "Robotic AND Hiatal Hernia AND Repair." The focus was on studies published from 2015 onwards. In total, 31 studies are included in this review.

Results: Evidence shows that robotic HH repair exhibits safety and efficacy levels comparable to those of the laparoscopic method across existing studies. Procedure duration varied depending on the surgeon’s experience with robotic tools, while the length of stay (LOS) at the hospital was often comparable to or occasionally shorter in robotic cases. Both approaches showed similar rates of postoperative complications and recurrences. Multiple studies indicated that robotic techniques resulted in lower blood loss and fewer transfusions, with one investigation highlighting reduced recovery times associated with robotic repair, especially for overweight and obese patients. However, robotic procedures consistently incurred higher costs. Notably, one study showed improved operative efficiency after seven to 15 surgeries as part of the learning curve.

Conclusions: Available literature indicates that robotic HH repair serves as a safe and practical alternative to laparoscopy, offering enhanced visualization and accuracy with similar outcomes. While there is encouraging data, the elevated costs and the scarcity of long-term randomized studies indicate a need for further investigation to identify the most effective surgical strategy.

## Introduction and background

The term 'hiatal hernia' (HH) describes the abnormal displacement of abdominal organs through the esophageal hiatus. These hernias are categorized into four types. Type I, or sliding, involves the upward movement of the gastroesophageal junction (GEJ) through the esophageal hiatus. Type II, or rolling, pertains to the herniation of the gastric fundus alone through the esophageal hiatus. Type III (a combination of sliding and rolling) consists of both the GEJ and gastric fundus protruding through the hiatus. And type IV includes the herniation of other intra-abdominal organs like the spleen, colon, and pancreas. Significantly, types II, III, and IV are collectively termed 'paraesophageal' hernias (PEH) [1].

Numerous techniques exist for HH repair, but there is no clear consensus on the best method. Traditionally, laparoscopic surgery has been regarded as the gold standard for treating symptomatic cases. However, laparoscopic HH repairs face significant hurdles, including high radiographic recurrence rates of 57% even at leading medical centers [2]. With advancements in surgical technology, the robotic approach is becoming increasingly common. This method has demonstrated improved recovery time and enhanced ergonomics for surgeons, with potential benefits including 3D visualization, greater magnification, improved dexterity, and instruments that can fully articulate [3]. Robotic systems are particularly advantageous for complex repairs, such as those for PEH [4]. While there is considerable research comparing open surgery with laparoscopy, studies contrasting laparoscopic techniques with robotic methods are scarce. This extensive review seeks to examine the existing literature on the technique, safety, and effectiveness of the robotic approach in HH repair, focusing on operative parameters and outcomes while comparing it to standard laparoscopic practices.

## Review

Materials and methods

The research was carried out between August 13 and October 1, 2025. We extensively searched the PubMed and Scopus databases twice with the specific keywords “Robotic AND Hiatal Hernia AND Repair.” To guarantee precision, thoroughness, accuracy, and completeness, we utilized a standardized data extraction form tailored to these keywords. Data extraction was undertaken manually. Information was collected through a unified data extraction form that included the specified terms. The study adhered to the Preferred Reporting Items for Systematic Reviews and Meta-Analyses Extension for Scoping Reviews (PRISMA-ScR) guidelines, which provide a systematic approach for executing scoping reviews [5]. All authors were involved in selecting studies, extracting data, and assessing the quality of the review.

The studies were meticulously chosen based on criteria that evaluated the robotic surgical repair of HH. This comprehensive review incorporated studies published between 2015 and 2025 to ensure relevance to current scientific discussions. Only articles in English specifically addressing the robotic method in HH repairs were included in the review. Our exclusion criteria were a) texts not in English, b) titles or abstracts irrelevant to the topic, c) studies not concentrating on HH and robotic surgery, and d) abstracts unavailable in the PubMed or Scopus databases.

**Figure 1:**
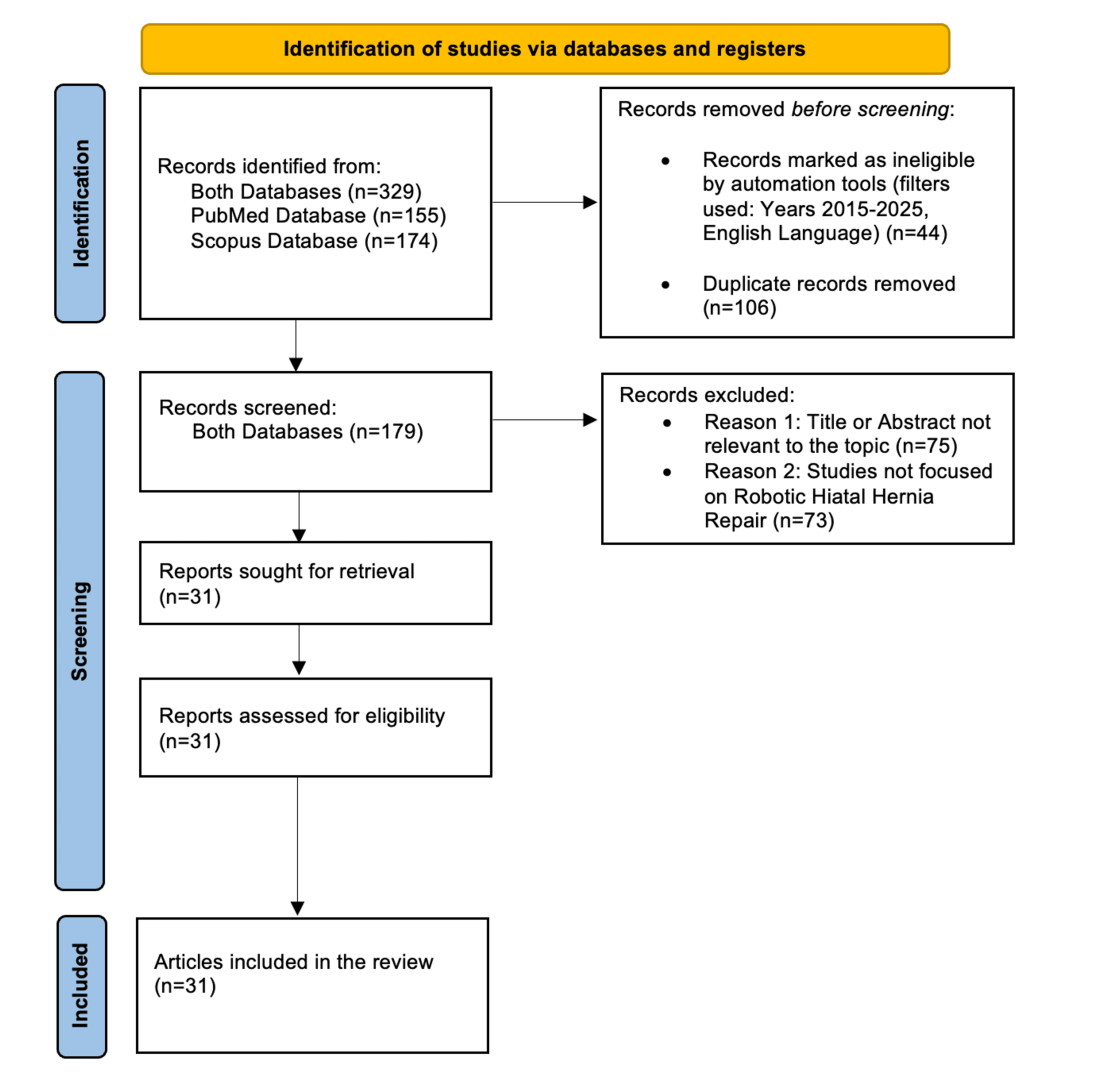
PRISMA diagram PRISMA: Preferred Reporting Items for Systematic Reviews and Meta-Analyses [5]

Following the PRISMA guidelines [5], we initially identified 155 records from PubMed and 174 from Scopus, totaling 329 records. We applied filters based on publication years (2015-2025) and the English language, leading to the exclusion of 44 records. Upon thorough review, we found 106 duplicates between the records from both databases. After excluding duplicates, we screened a total of 179 unique records derived from both databases, leading to the rejection of 148 articles. The exclusions were primarily due to two reasons: 75 articles had titles or abstracts unrelated to the topic, and 73 studies did not focus on HH and robotic surgery, making them irrelevant to our research. Thus, 31 reports were sought for retrieval and subsequently assessed for eligibility. As all 31 articles were considered eligible, this article is based on data from these 31 credible sources (Figure 1).

A rigorous methodology was employed to assess the data in line with the categorization outlined above. In developing themes, we considered the robotic approach to HH repair by analyzing operative parameters and comparing them with those of standard laparoscopic methods, as presented in the articles cited. Table 1 summarizes the characteristics of the included studies. Two authors independently conducted the literature screening of the articles.

**Table 1:** Characteristics of the included studies HH: Hiatal hernia, RAHHR: Robot-assisted hiatal hernia repair, OR: Operative room, PEH: Paraesophageal hernia, PEHR: Paraesophageal hernia repair, RPEHR: Robotic paraesophageal hernia repair, LOS: Length of stay, cUDS: Complete upside-down stomach, cUDSH: Complete upside-down stomach hernia, RYGB: Roux-en-Y gastric bypass, SG: Sleeve gastrectomy, PE: Pulmonary embolism, Early experience group: Procedures performed within the first year of introduction of robotic technique, Late experience group: Procedures performed in the second year, NM: Not mentioned

Author	Total no. of patients	No. of patients undergoing RAHHR	Study type	Hernia type	OR time (minutes)	LOS (days)	Postoperative complications	Reoperation	Cost	Key findings
Obisesan et al. [1]	112	112	Retrospective	All	216	1.8	NM	NM	NM	Pulmonary embolism (PE) events following robot-assisted hiatal hernia repair (RAHHR), although uncommon, are associated with increased LOS and costs.
Gerull et al. [2]	233	233	Prospective	Paraesophageal hernia (PEH) II (3); III (181); IV (49)	183.8	1.9	NM	0.5% (1/233)	NM	Results demonstrate that robotic PEH repair with an experienced surgical team is a safe and effective alternative to laparoscopic repair, with excellent long-term outcomes, including a very low recurrence rate.
O'Connor et al. [3]	392	114	Retrospective	All	179	2.3	NM	NM	NM	RAHHR offers technical advantages over laparoscopic repair with similar clinical outcomes.
Vasudevan et al. [4]	28	28	Retrospective	PEH II (3); III (22); IV(3)	83.6	2.8	10.7%(3/28)	NM	NM	Robotic paraesophageal hernia repair (RPEHR) is a safe and effective procedure with acceptable complication rates even in older patients with high operative risks.
Sadeghi et al. [6]	144	144	Retrospective	All I (89); II (23); III (30); IV (2)	173	2	18%(26/144)	4.2%(6)	NM	RAHHR has similar operative times, LOS, and complications compared to laparoscopic repair.
Straatman et al. [7]	109	109	Retrospective	All	113	1	NM	13.8%(15/109)	NM	The learning curve for robotic HH repair may be as little as seven to 15 cases.
Washington et al. [8]	30	Early experience (13); Late experience (17)	Retrospective	PEH	184 (early experience); 142 (late experience)	NM	NM	NM	NM	In the early experience robotic HH repair group, procedures can be more difficult than expected, even in the hands of an experienced laparoscopic team.
Sarkaria et al. [9]	24	24	Retrospective	Giant PEH	277	4	50% (12/24)	NM	NM	Robot-assisted giant paraesophageal hernia repair (PEHR) is safe, with reported short-term, operative, and functional outcomes similar to conventional laparoscopic approaches.
Han et al. [10]	NM	NM	Pilot study	NM	NM	NM	NM	NM	NM	A novel pilot curriculum for robotic HH repair was developed for senior surgical residents to provide training. Results indicate that live-operative robotic training is not sufficient alone for advanced robotic skill training. The addition of simulations is encouraged.
Lekarczyk et al. [11]	73	31	Retrospective	All (4); II (2); III (23); IV (2)	257	2	NM	NM	15,043$	Despite higher supply costs and charges for RAHHR, hospital profits were similar when compared to laparoscopic approaches. Short-term clinical outcomes were also similar.
Munshower et al. [12]	NM	NM	Retrospective	NM	NM	NM	NM	NM	6,852.41$ (No mesh); 7,511.09$ (with mesh)	PEH repairs without mesh had no significant difference in overall cost. However, in RPEHR with mesh, the cost was significantly higher compared to the laparoscopic group.
Gerull et al. [13]	1,854	830	Retrospective	PEH II (15); III (659); IV (156)	174.1	1.8	NM	0.2% (2/830)	2,147$	Findings show that robotic PEH repair is safe and can result in improved perioperative outcomes.
Perrone et al. [14]	20	20	Retrospective	All	166	2	NM	NM	NM	RAHHR with Collis gastroplasty showed increased OR time and LOS compared to repair without Collis gastroplasty.
Soliman et al. [15]	293	142	Retrospective	All I (50); II (0); III (87); IV (5)	186.5	1.3	6.3% (9/142)	20.4% (29/144)	NM	Robotic surgery had improved outcomes compared to laparoscopic HH repair, despite a higher incidence of reoperative cases in the robotic group.
Ward et al. [16]	207	90	Retrospective	PEH II (2); III (83); IV (5)	178	2	17% (15/90)	3% (3/90)	NM	RPEHR had shorter LOS and fewer complications than transthoracic.
Saleh et al. [17]	297	193	Retrospective	NM	NM	NM	NM	NM	NM	Presented a novel critical view technique that results in favorable postoperative outcomes, including a reduction in recurrence and reoperation in PEH repairs.
Tonelli et al. [18]	2,444	178	Retrospective	All	250.2	NM	7% (12/178)	4% (7/178)	NM	Repairs done robotically took longer but had 30-day outcomes and rates of reoperation for recurrence equivalent to laparoscopic repairs.
Benedix et al. [19]	140	55	Retrospective	All	149	3.6	10.9% (6/55)	1.8% (1/55)	NM	Results failed to demonstrate a clear advantage of the robotic technique in patients with refractory gastroesophageal reflux disease and/or symptomatic HH.
Howell et al. [20]	128	44	Retrospective	All I (2); II (4); III (36); IV (1)	NM	2	13.6% (6/44)	4.6% (2/44)	NM	Minimally invasive HH repair is safe and feasible. The use of mesh-reinforced crural repair does not adversely affect short-term outcomes such as 30-day readmission but may trend towards an increased rate of short-term complications.
Sowards et al. [21]	298	Primary PEH (247); recurrent PEH (51)	Retrospective	PEH primary; recurrent	116 (primary); 160 (recurrent)	1.3 (primary); 1.9 (recurrent)	NM	2%( 6) primary; 6% (3) recurrent	NM	Recurrent RPEHR repairs have similar perioperative and postoperative outcomes compared to primary PEH repairs.
Mertens et al. [22]	362	362	Retrospective	All	148	3	14% (52/362)	NM	NM	Serious morbidity was estimated at 5.2% for primary surgery and 2.6% for redo surgery.
Kirkpatrick et al. [23]	7	7	Retrospective	All	138	NM	NM	NM	NM	This review shows that the use of the robot has proven to be safe and effective, having many benefits in HH repair. Robotics allows us to perform minimally invasive hernia repairs on large and complex defects that would have otherwise been performed via an open approach.
Galvani et al. [24]	61	61	Retrospective	PEH II (26); III (64); IV (13)	186	2.6	22.9% (14/61)	NM	NM	RPEHR has proved to be feasible and safe, with a learning curve comparable to the standard laparoscopic approach.
Bassiri et al. [25]	8,019	2,986	Retrospective	NM	NM	NM	NM	NM	NM	Robotic HH repair was associated with superior outcomes in reducing postoperative ileus, ICU visits, one-year symptom recurrence, and endoscopic intervention compared with the laparoscopic approach, suggesting its superiority in minimally invasive hiatal hernia repair.
Sebastian et al. [26]	75,034	4,639 sleeve gastrectomy (SG); 1,502 Roux-en-Y gastric bypass (RYGB)	Retrospective	All	102.31 (SG); 163.48 (RYGB)	1.52 (SG); 2 (RYGB)	NM	1,03% (48/4639) SG; 2,7%(40/1502) RYGB	NM	Robotic concurrent bariatric surgery and HH repair leads to similar overall clinical outcomes as the laparoscopic approach despite longer operative times. Furthermore, the robotic approach is associated with reduced blood transfusion and anastomotic leak incidence in the RYGB group.
Knewitz et al. [27]	52	31	Retrospective	PEH	233.9	2.67	NM	NM	NM	Minimally invasive PEH repair and RYGB is a feasible and effective procedure.
Ward et al. [28]	168,329	9,897	Retrospective	PEH	NM	NM	NM	12.6%	NM	Robotic PEH repair is associated with significantly more complications compared to laparoscopic paraesophageal hernia repair, even in high-volume centers.
Rebibo et al. [29]	1	1	Case report	Giant HH	150	3	NM	NM	NM	The patient was able to resume oral feeding on the second postoperative day and was discharged home on the third postoperative day.
Ceccarelli et al. [30]	5	3	Case series	Giant HH	NM	NM	33% (1/3)	NM	NM	The robot-assisted approach, allowing a stable 3D view and the use of articulated instruments, represents a reasonable option in challenging situations.
Wilhelm et al. [31]	55	36	Prospective	cUDSH	232	8.5	36% (13/36)	0% (0)	NM	While robot-assisted surgery provides additional precision, enhanced visualization, and greater feasibility in complete upside-down stomach (cUDS) HH repair, clinical outcomes are at least equal to those obtained by standard laparoscopic surgery.
Liu et al. [32]	142	Normal BMI (10); overweight (7); obese (13)	Retrospective	All	113 (normal); 115 (overweight); 120 (obese)	5 (normal); 4 (overweight); 4 (obese)	NM	NM	¥52,529 (normal); ¥62,453 (overweight); ¥62,916 (obese)	Under the financial burden of the higher costs associated with robot-assisted surgery, choosing robot-assisted surgery can provide more benefits for patients with a higher BMI (overweight or obese).

Results

We conducted an extensive review of 31 articles published from 2015 to 2025, utilizing the PubMed and Scopus databases. The majority of the selected studies were retrospective cohort studies, with the exceptions of two prospective studies (Wilhelm et al. and Gerull et al.), one case series (Ceccarelli et al.), and one case report (Rebibo et al.) [31,2,30,29]. Following a rigorous analysis of these documents, we concluded that the use of robotic techniques for HH repair presents a viable alternative to conventional laparoscopic surgery, demonstrating safety and generally comparable intraoperative and postoperative outcomes. In certain cases, robotic surgeries may even yield superior results compared to traditional laparoscopic methods.

Operative times tended to vary; some studies indicated a longer average duration with robotic assistance (for instance, Tonelli et al. [18] reported 4.17 hours versus 3.57 hours), while others noted shorter times (e.g., Gerull et al. [2] found 174.1 minutes versus 187.3 minutes). The LOS was also found to vary, but typically aligned closely with laparoscopic repair durations. Some studies (e.g., O'Connor et al. [3] noted LOS of 2.2 days compared to 3.3 days) demonstrated a significantly shorter LOS with robotic approaches.

Most studies indicated comparable postoperative complications between robotic and laparoscopic methods. One investigation (Sebastian et al. [26]) showed that in a specific cohort (Roux-en-Y gastric bypass (RYGB)), robotic procedures led to reduced blood transfusion rates (0.3% vs. 1.7%), lower instances of postoperative bleeding (0.4% vs. 1.1%), and fewer anastomotic/staple line leaks (0.2% vs. 0.8%) compared to laparoscopic surgeries. The study by Gerull et al. [2] highlighted the advantages of robotic techniques in terms of estimated blood loss (EBL), which was significantly lower (27.3 mL vs. 89.3 mL). However, one study (Ward et al. [16]) noted an increased risk of complications with robotic PEH repair, even in high-volume centers (OR (95% CI) = 1.17 (1.07, 1.27)). The rates of reoperation due to recurrence were comparable between the two surgical methods.

A crucial consideration is the cost associated with robotic equipment. Lekarczyk et al. [11] concluded that while robotic surgery incurs higher overall costs, it results in similar hospital profits compared to laparoscopic techniques. Given these findings and considering the financial implications of robotic equipment, robotic HH repair may be particularly advantageous for patients with elevated BMI, specifically those classified as overweight or obese. Liu et al. [32] indicated that in individuals with a BMI in the range of 24 to < 28.0 kg/m² or ≥ 28.0 kg/m², robotic surgery was linked to significantly reduced intraoperative bleeding, shorter postoperative time to first flatus, decreased hospitalization duration, and lower pain scores. In patients with normal BMI (18.5 to <24.0 kg/m²), robotic-assisted surgery showed no notable differences in terms of postoperative hospitalization duration or pain scores.

Discussion

The aim of this extensive review was to investigate the existing literature concerning the use of robotics in HH repair while comparing it to traditional laparoscopic methods. We emphasized key data about overall operative duration, LOS, the entirety of postoperative complications, and rates of reoperation due to recurrence. The cost of robotic equipment plays a crucial role when assessing patients with HH requiring surgery. Generally, our results indicate that robotic surgery serves as a safe and viable alternative, presenting certain potential benefits, although conclusive statements are somewhat restricted by the quality and variability of current studies.

As previously noted, the majority of studies assessed were retrospective cohort analyses. To our knowledge, no randomized controlled trials (RCTs) have been conducted on this specific topic, which must be considered when determining the robustness and reliability of the conclusions drawn. The specific type of HH discussed in each article must also be taken into account. Of the 31 articles, 11 focused solely on PEH, two dealt exclusively with giant HH, and one concentrated on complete upside-down stomach (cUDS) hernias. The remaining 17 covered all four classic types (I-IV) of HH.

Mesh is commonly utilized in HH repairs, and national guidelines recommend its use for larger hernias to reduce short-term recurrence rates. There is scant evidence involving robotic HH repairs performed without mesh. One study (Sadeghi et al. [6]) highlighted the serious problems associated with hiatal mesh, particularly esophageal erosion, which has an incidence of about 5%. They proposed a new robotic technique for HH repair that avoids mesh by closing the hiatus with a mix of absorbable and non-absorbable barbed sutures, resulting in lower recurrence rates and reduced mesh-related complications. The robot enhances precision and control, especially during esophageal mobilization, suture placement on the hiatus, and fundoplication. This study demonstrated that the robotic method is both safe and feasible while presenting an alternative approach without mesh [6]. While mesh-free techniques appear as an alternative, the evidence supporting their superiority in reducing recurrence for large hernias is still limited compared to the standard practice.

The learning curve associated with robotic procedures must also be discussed. Several studies indicated improved outcomes as surgeons gained experience with robotics. Straatman et al. suggested that the learning curve for robotic fundoplication could be as few as seven to 15 cases within a structured learning program featuring proctoring [7]. Washington et al. divided 30 consecutive patients over two years into early and late experience groups, finding statistically significant differences in mean operative times (184 minutes versus 142 minutes) and in the number of conversions to open surgery (4 versus 0 patients) between the groups. Influencing factors could include inappropriate patient positioning, inexperience of surgical staff, and limited technical experience of the surgeon with robotic systems [8]. Sarkaria et al. observed a decrease in median procedure times, initially recorded at 277 minutes (range: 185-485), as experience increased [9]. A pilot study (Han et al.) recommended introducing simulations to enhance both technical and non-technical skills in robotic surgery [10].

The broad adoption of robotic systems in clinical practice may be impeded by the associated costs of robotic equipment. One study (Lekarczyk et al.) noted that when comparing robotic and laparoscopic approaches for HH repair, factors such as LOS and operative time showed no significant difference. However, robotic methods were linked to higher supply costs and patient charges, with no notable difference in hospital profit between the two procedures (robotic versus laparoscopic) [11]. Conversely, Munshower et al. found that the cost of the robotic approach for PEH repair exceeded that of laparoscopic methods when mesh was utilized (without mesh, costs were similar) [12]. Interestingly, Gerull et al. [2] reported similar OR equipment costs for both robotic and laparoscopic PEH procedures, which contrasts with typical findings that associate robotics with higher overall expenses. A potential explanation is that while individual robotic instruments cost more than laparoscopic ones, the overall expenditure may balance as certain laparoscopic cases necessitate conversion to open surgery and additional stapling tools for esophageal lengthening [13].

Overall, outcomes appear to be comparable between robotic and laparoscopic HH repairs, albeit with variation among individual studies. For instance, Gerull et al. suggested that the robotic technique may offer unique advantages for mediastinal mobilization [2]. The mediastinum is often regarded as one of the most challenging anatomical areas to access, and procedures such as Collis gastroplasty are frequently required to achieve effective mobilization through laparoscopic techniques. These often correlate with poor postoperative outcomes, including increased GERD symptoms and dysphagia. Robotics affords enhanced access to the mediastinum, aided by the extended length of robotic instruments, which facilitates retraction and exposure of the hiatus alongside distal end wrists that promote circumferential dissection [2,13,14].

O’Connor et al. observed that patients undergoing robotic repairs exhibited lower rates of radiographic recurrence after one year, even among those with initial repairs for recurrence. Reoperative surgeries often face complications due to dense adhesions around structures such as the esophagus and pleura, which raise the likelihood of failure. The robotic technique enhances visualization and dexterity, helping to preserve the crura and allowing for precise suturing despite the challenges posed by adhesion. This method also minimizes collateral damage to surrounding structures, effectively reducing LOS [3]. Likewise, Soliman et al. found improved outcomes in the robotic cohort despite an increase in reoperative rates [15].

Current literature indicates that robotic methods can be safely executed, presenting a low and acceptable incidence of both short- and long-term complications [4,16-24]. Bassiri et al. even reported superior results for robotic HH surgery, particularly in reducing the incidence of postoperative ileus, ICU admissions, and recurrence of symptoms one year post-surgery, necessitating endoscopic intervention [25]. One study (Sebastian R. et al.) examined the outcomes of robotic versus laparoscopic repairs alongside concurrent bariatric surgery, confirming the feasibility of combining these approaches, especially in the RYGB group, where the robotic method led to decreased blood transfusions and anastomotic leaks. The enhanced hemostatic control attributed to robotic techniques, characterized by precise movements within a 3D magnified surgical field, is believed to play a significant role [26]. Knewitz et al. also found that performing primary RYGB alongside robotic PEH repair is a secure option, potentially resulting in shorter operative durations compared to laparoscopy (233.90 min vs. 261.52 min) [27].

In terms of complications, a large retrospective study involving 168,329 participants (Ward et al.) indicated that the overall adjusted complication rate was significantly higher in patients undergoing robotic PEH repairs compared to laparoscopic repairs (OR (95% CI) = 1.17 (1.07, 1.27)). Furthermore, rates of respiratory failure (OR (95% CI) = 1.68 (1.37, 2.05)) and esophageal perforation (OR (95% CI) = 2.19 (1.42, 3.93)) were elevated in the robotic cohort, two of the most challenging complications to manage [28]. Pulmonary embolism (PE), a known serious postoperative complication, is a risk during robotic procedures due to pneumoperitoneum, causing decreased venous return to the heart, leading to venous stasis. However, existing literature does not indicate a higher incidence of PE following robotic HH repairs compared to open surgeries [1].

Several specific subtypes of HH can also be repaired using robotic techniques, as documented in the literature. A giant HH, wherein a significant portion of the stomach herniates into the thoracic cavity, could benefit from robotics, especially in emergencies [29,30]. For surgeons skilled in laparoscopic repairs of gHH, transitioning to robot-assisted methods is considered a relatively short learning curve [9]. The cUDS hernias present critical clinical challenges due to their life-threatening potential. In a comparative study of robot-assisted versus laparoscopic repair for cUDS (Wilhelm et al.), outcomes were found to be at least equivalent, affirming the viability of the robotic approach [31]. 

Liu et al. conducted a unique study that assessed the efficacy of robotic and laparoscopic HH repair while stratifying patients based on BMI. They found that in overweight and obese patients, the robotic approach was associated with significant differences in hospitalization duration (4.0 versus 5.0 days for the robotic and laparoscopic approaches, respectively) and lower postoperative pain scores that were not observed in the normal BMI group. Perhaps, in overweight and obese patients, increased fat (subcutaneous and visceral) makes anatomical dissection more challenging. Moreover, the dynamic characteristics of the hiatus region pose challenges during suturing. These problems can be managed due to the robotic system’s 3D imaging and comprehensive field of view, enhanced stability, and reduced risk of operating errors that stem from its ergonomic design [32].

Despite the comprehensive nature of this scoping review, several limitations should be acknowledged. As mentioned before, the majority of the included studies were retrospective studies. Only a few were prospective, and none were RCTs, ultimately mitigating the validity of the evidence and increasing the risk of bias. Moreover, direct comparability of study results is limited by significant heterogeneity among the included studies regarding patient populations, surgeon experience, hernia classification and types, operative technique, and measured outcomes. It should be noted that there is a scarcity of long-term follow-up data. The exclusion of non-English publications might have also contributed to bias in the observed results. Future research should prioritize well-designed RCTs with standardized reporting criteria to validate and expand upon the findings of this review.

## Conclusions

Overall, advancements in technology have facilitated the use of robotic tools and instruments in various surgical procedures, including HH repair. Although robotic systems offer distinct benefits in certain contexts, there is no definitive agreement on the best method to adopt. Existing research indicates that the robotic approach may serve as a viable substitute for the laparoscopic technique. Nonetheless, factors like the learning curve experienced during the shift to the robotic system and the high expenses associated with robotic technology should be thoroughly considered when deciding on the appropriate method to use. Future studies should include comparisons of different robotic systems (e.g., Da Vinci Xi versus Da Vinci 5 (DV5); Intuitive Surgical Inc., Sunnyvale, CA, USA) to assess whether different robotic systems impact the outcomes of these procedures.

## References

[1] Obisesan A, Singhal V, Satoskar S (2022). Robotic-assisted hiatal hernia repair and pulmonary embolism: an institution-based retrospective cohort study. J Robot Surg.

[2] Gerull WD, Cho D, Kuo I, Arefanian S, Kushner BS, Awad MM (2020). Robotic approach to paraesophageal hernia repair results in low long-term recurrence rate and beneficial patient-centered outcomes. J Am Coll Surg.

[3] O'Connor SC, Mallard M, Desai SS (2020). Robotic versus laparoscopic approach to hiatal hernia repair: results after 7 years of robotic experience. Am Surg.

[4] Vasudevan V, Reusche R, Nelson E, Kaza S (2018). Robotic paraesophageal hernia repair: a single-center experience and systematic review. J Robot Surg.

[5] Tricco AC, Lillie E, Zarin W (2018). PRISMA Extension for Scoping Reviews (PRISMA-ScR): checklist and explanation. Ann Intern Med.

[6] Sadeghi JK, Li LT, Singh VA (2024). Robotic hiatal hernia repair without mesh. J Thorac Dis.

[7] Straatman J, Rahman SA, Carter NC, Mercer SJ, Knight BC, van Boxel GI, Pucher PH (2023). Proctored adoption of robotic hiatus hernia surgery: outcomes and learning curves in a high-volume UK centre. Surg Endosc.

[8] Washington K, Watkins JR, Jeyarajah DR (2020). The first year is the hardest: a comparison of early versus late experience after the introduction of robotic hiatal hernia repair. J Robot Surg.

[9] Sarkaria IS, Latif MJ, Bianco VJ, Bains MS, Rusch VW, Jones DR, Rizk NP (2017). Early operative outcomes and learning curve of robotic assisted giant paraesophageal hernia repair. Int J Med Robot.

[10] Han BJ, Sherrill W 3rd, Awad MM (2023). The use of advanced robotic simulation labs to advance and assess senior resident robotic skills and operating room leadership competency: a pilot study. Surg Endosc.

[11] Lekarczyk A, Sinha H, Dvir D, Goyert J, Airhart A, Reddy RM (2023). Similar hospital profits with robotic-assisted paraesophageal hiatal hernia repair, despite higher or supply costs. Surg Endosc.

[12] Munshower E, Ren E, Bauerle WB (2023). Cost analysis of robotic assisted general surgery cases in a single academic institution. J Robot Surg.

[13] Gerull WD, Cho D, Arefanian S, Kushner BS, Awad MM (2021). Favorable peri-operative outcomes observed in paraesophageal hernia repair with robotic approach. Surg Endosc.

[14] Perrone JA, Yee S, Guerrero M, Wang A, Hanley B, Zuberi J, Damani T (2022). Comparative analysis of patients with robotic hiatal hernia repairs with and without Collis gastroplasty. Am Surg.

[15] Soliman BG, Nguyen DT, Chan EY, Chihara RK, Meisenbach LM, Graviss EA, Kim MP (2020). Robot-assisted hiatal hernia repair demonstrates favorable short-term outcomes compared to laparoscopic hiatal hernia repair. Surg Endosc.

[16] Ward KR, Bui J, Bondarenko I (2025). Improved outcomes with robotic-assisted laparoscopic paraesophageal hernia repairs compared with laparoscopic and transthoracic approaches: a single high-volume institution experience. JTCVS Open.

[17] Saleh Z, Verchio V, Ghanem YK (2024). Optimizing outcomes in paraesophageal hernia repair: a novel critical view. Surg Endosc.

[18] Tonelli CM, Baker MS, Luchette FA, Cohn T (2023). Laparoscopic and robotic paraesophageal hernia repair in United States veterans: clinical outcomes and risk factors associated with reoperation recurrence. Am J Surg.

[19] Benedix F, Adolf D, Peglow S, Gstettenbauer LM, Croner R (2021). Short-term outcome after robot-assisted hiatal hernia and anti-reflux surgery-is there a benefit for the patient?. Langenbecks Arch Surg.

[20] Howell RS, Liu HH, Petrone P (2020). Short-term outcomes in patients undergoing paraesophageal hiatal hernia repair. Sci Rep.

[21] Sowards KJ, Holton NF, Elliott EG (2020). Safety of robotic assisted laparoscopic recurrent paraesophageal hernia repair: insights from a large single institution experience. Surg Endosc.

[22] Mertens AC, Tolboom RC, Zavrtanik H, Draaisma WA, Broeders IA (2019). Morbidity and mortality in complex robot-assisted hiatal hernia surgery: 7-year experience in a high-volume center. Surg Endosc.

[23] Kirkpatrick T, Zimmerman B, LeBlanc K (2018). Initial experience with robotic hernia repairs: a review of 150 cases. Surg Technol Int.

[24] Galvani CA, Loebl H, Osuchukwu O, Samamé J, Apel ME, Ghaderi I (2016). Robotic-assisted paraesophageal hernia repair: initial experience at a single institution. J Laparoendosc Adv Surg Tech A.

[25] Bassiri A, Pawar OS, Boutros C (2025). Robotic vs laparoscopic hiatal hernia repair: a comparative study of short- and long-term surgical outcomes. Ann Thorac Surg.

[26] Sebastian R, Ghanem OM, Cornejo J, Ruttger T, Mayuiers M, Adrales G, Li C (2022). Robot-assisted versus laparoscopic approach to concurrent bariatric surgery and hiatal hernia repair: propensity score matching analysis using the 2015-2018 MBSAQIP. Surg Endosc.

[27] Knewitz D, Aguilar JC, Fullerton S, Evans L, Bowers S, Elli E (2025). Primary Roux-en-Y gastric bypass with concurrent paraesophageal hernia repair in obese patients. JSLS.

[28] Ward MA, Hasan SS, Sanchez CE, Whitfield EP, Ogola GO, Leeds SG (2021). Complications following robotic hiatal hernia repair are higher compared to laparoscopy. J Gastrointest Surg.

[29] Rebibo L, Boutron C, Msika S (2020). Robotic-assisted hiatal hernia repair with prosthetic reinforcement (with video). J Visc Surg.

[30] Ceccarelli G, Pasculli A, Bugiantella W (2020). Minimally invasive laparoscopic and robot-assisted emergency treatment of strangulated giant hiatal hernias: report of five cases and literature review. World J Emerg Surg.

[31] Wilhelm A, Nocera F, Schneider R (2022). Robot-assisted vs. laparoscopic repair of complete upside-down stomach hiatal hernia (the RATHER-study): a prospective comparative single center study. Surg Endosc.

[32] Liu M, Li C, Yao J (2025). Body mass index should be considered as an indicator for laparoscopic surgery or robot-assisted surgery selection in patients with hiatal hernia. J Thorac Dis.

